# Transient Peripapillary Retinoschisis in Glaucomatous Eyes

**DOI:** 10.1155/2017/1536030

**Published:** 2017-01-12

**Authors:** Josine van der Schoot, Koenraad A. Vermeer, Hans G. Lemij

**Affiliations:** ^1^Rotterdam Ophthalmic Institute, The Rotterdam Eye Hospital, Rotterdam, Netherlands; ^2^Glaucoma Service, The Rotterdam Eye Hospital, Rotterdam, Netherlands

## Abstract

*Purpose*. To investigate transient focal microcystic retinoschisis in glaucomatous eyes in images obtained with several imaging techniques used in daily glaucoma care.* Methods*. Images of 117 glaucoma patients and 91 healthy subjects participating in a large prospective follow-up study into glaucoma imaging were reviewed. Participants were measured with spectral domain optical coherence tomography (SD-OCT), scanning laser polarimetry (SLP), scanning laser tomography (SLT), and standard automated perimetry (SAP). The presence of a focal retinoschisis in SD-OCT was observed and correlated to SLP, SLT, and SAP measurements, both cross-sectionally and longitudinally.* Results*. Seven out of 117 glaucoma patients showed a transient, localised, peripapillary, heterogeneous microcystic schisis of the retinal nerve fiber layer (RNFL) and sometimes other retinal layers as well in SD-OCT. None of the healthy eyes showed this phenomenon nor did any of the other imaging techniques display it as detailed and consistently as did the SD-OCT. SAP showed a temporarily decreased focal retinal sensitivity during the retinoschisis and we found no signs of glaucomatous progression related to the retinoschisis.* Conclusions*. Transient microcystic retinoschisis appears to be associated with glaucomatous wedge defects in the RNFL. It was best observed with SD-OCT and it was absent in healthy eyes. We found no evidence that the retinoschisis predicted glaucomatous progression.

## 1. Introduction

In glaucoma, retinal nerve fibers are damaged and lost, leading to thinning of the retinal nerve fiber layer (RNFL) [1, 2]. The thinning of the RNFL can be measured by different imaging techniques, such as scanning laser polarimetry (SLP), scanning laser tomography (SLT), and optical coherence tomography (OCT) [3]. The current generation OCT, that is, spectral domain OCT (SD-OCT) [4–7], shows a more detailed image of all layers of the retina compared to time domain OCT, which potentially leads to new insights.

Several recent studies presented cases with peripapillary retinoschisis, imaged with SD-OCT [8–13]. All studies showed a relation of the retinoschisis with the presence of glaucoma. Kahook et al. [11] and Lee et al. [12] related the retinoschisis also to high or fluctuating intraocular pressures. Following a large population for several years, Lee et al. were able to observe spontaneous resolution of the retinoschisis. Recently, Lee et al. presented alterations of the lamina cribrosa to be associated with peripapillary retinoschisis [13]. To date, several insights in this newly described phenomenon have been presented, but the pathogenesis and the course of the retinoschisis remain uncertain. Also, whether the retinoschisis is related to glaucoma progression and whether it can also be imaged by other techniques used in daily glaucoma care still are unknown.

In the current study, we describe the appearance of this peripapillary retinoschisis over time that we observed in SD-OCT images of a subset of subjects participating in a large, prospective longitudinal study on glaucoma imaging. We also explore the effects of the retinoschisis on the measurements of various other, commonly used functional and structural techniques in glaucoma care. In addition, we assess a potential correlation between the appearance or disappearance of the retinoschisis and glaucomatous progression, measured by several techniques.

## 2. Methods

### 2.1. Participants

This report presents the outcome of reviewing a study cohort of 117 glaucoma patients and 91 subjects with healthy eyes on the presence of a peripapillary retinoschisis. All subjects participated in a longitudinal prospective follow-up study into glaucoma imaging. All acquired images that had been taken in 4 successive years were retrospectively reviewed for the presence of any peripapillary retinoschisis.

To be qualified as healthy, a subject was required to have a normal visual field and an intraocular pressure of 22 mmHg or less. A normal visual field had, by definition, a Glaucoma Hemifield Test within normal limits and a Mean Deviation (MD) and Pattern Standard Deviation (PSD) within their respective 95% confidence intervals. Visual fields were considered as glaucomatous if at least two of the following findings were confirmed on the successive visual field: a PSD significant at the 5% probability level, a Glaucoma Hemifield Test outside normal limits, and a cluster of 3 or more points below the 5% probability level or 1 individual point below the 1% probability level.

Subjects were excluded from participation in the presence of any significant coexisting ocular or systemic disease known to possibly affect the visual field (e.g., diabetes mellitus), a history of intraocular surgery (except for uncomplicated cataract surgery and glaucoma surgery in glaucoma patients), uncontrollable arterial hypertension, and secondary glaucoma.

All participants had a best-corrected Snellen visual acuity of at least 20/40 in both eyes, a spherical equivalent refractive error between −10.0 D and +5.0 D, and unremarkable findings upon slit-lamp examination, including open angles on gonioscopy.

All glaucoma patients were recruited at the Glaucoma Service at the Rotterdam Eye Hospital. The healthy subjects were mainly friends and spouses of the glaucoma patients who volunteered to participate in the longitudinal study. The methods and procedures used in this study adhered to the Declaration of Helsinki. Informed consent was obtained from all participants. This study was approved by the Medical Ethics Committee of the Erasmus Medical Centre, Rotterdam, Netherlands.

### 2.2. Image Acquisition

All participants were measured with the Spectralis OCT (Heidelberg Engineering GmbH, Dossenheim, Germany), an imaging device that features SD-OCT. With SD-OCT, the peripapillary areas of both eyes were scanned by means of a volume scan of 20 by 20 degrees.

In the same session, all participants were measured with several other imaging techniques as well, including SLP (GDxECC [GDx], Carl Zeiss Meditec Inc., Dublin, CA, USA) and SLT (Heidelberg Retina Tomograph III [HRT], Heidelberg Engineering GmbH, Dossenheim, Germany). All participants also underwent visual field testing (Humphrey Field Analyzer [HFA], Carl Zeiss Meditec Inc., Dublin, CA, USA). All measurements were repeated every six months in glaucomatous cases and every 12 months in healthy cases.

### 2.3. Analysis

#### 2.3.1. Cross-Sectional

Because we incidentally noticed a focal schisis of the RNFL and sometimes of other retinal layers as well in some of the SD-OCT images, we systematically reviewed all images of all participants in the longitudinal study to detect a similar schisis in other eyes. The cases that showed the focal retinoschisis were followed over time to evaluate its course. We also explored whether any of the other imaging devices allowed us to image the schisis. In addition, we compared the area of schisis, measured with SD-OCT, to the corresponding sectors of the visual field, using the method introduced by Garway-Heath et al. [14] To indicate the presence and size of a glaucomatous defect, we counted the number of points below the 0.5% probability level within the corresponding sector in either the total or the pattern deviation probability plot, whichever showed the fewest abnormal points. In the GDx, we examined the retardation map. The HRT has no specific parameters to quantify RNFL thickness around the optic nerve head (ONH). Therefore, the three-dimensional topography image (HRT-3D) was the only way in the HRT to quantitatively determine the surface height around the ONH.

To explore whether our observation might be related to retinal disease, such as posterior vitreous detachment (PVD), we retrospectively reviewed all SD-OCT images containing the RNFL schisis. Peripapillary vitreoretinal traction syndrome, with retinal thickening next to the ONH is associated with PVD [15, 16]. We therefore explored the presence of other retinal diseases by careful scrutiny of all SD-OCT B-scans at large magnification.

#### 2.3.2. Longitudinal

When possible, we compared the images during the focal retinoschisis with the outcome either before or after the schisis. In the VF, we calculated the mean retinal sensitivity (MRS) of all individual points in the corresponding sector, as presented on the printout. We used the paired Student *t*-test to evaluate differences between the MRS of the measurements during and before/after the disturbed area. In the HRT, Topographic Change Analysis (HRT-TCA) was used to determine any changes in the height of the RNFL.

One hundred and eighty-nine eyes of 117 glaucoma patients (66 men, 51 women), as well as 182 eyes of 91 healthy subjects (36 men, 55 women), were measured at least once, with a maximum follow-up time of 34 months. The mean age of the healthy subjects was 57 years (SD 13) and of glaucoma patients 69 years (SD 10).

## 3. Results

Seven out of 189 glaucomatous eyes showed a transient, localised, peripapillary schisis of the RNFL and sometimes of other retinal layers as well. This phenomenon was not observed in any of the 182 healthy eyes (*p* = 0.0089, *χ*^2^-test). All 7 subjects were women. Their mean age was 70.7 years (SD 8.3). The subject and scan characteristics have been presented in Tables 1 and 2.

### 3.1. Scan Images

**Figure 1:**
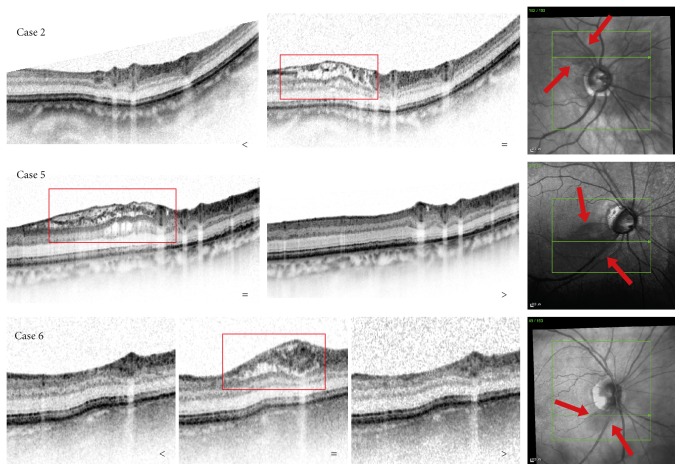
IR (right) and SD-OCT images of 3 cases, representing cases with measurements before (<) and during (=) the retinoschisis (Case 2) and representing cases with measurements during and after (>) the retinoschisis (Case 5). Case 6 was the only case imaged before (<), during (=), and after (>) the presence of the retinoschisis. The detail inside the red boxes represents the retinoschisis. Between the red arrows in the IR fundus images is the area of the focal retinoschisis. IR: infrared image; SD-OCT: spectral domain optical coherence tomography.

The SD-OCT scans of all 7 cases showed an area of schisis of the RNFL. This area was well demarcated on the en-face infrared image and it verged on the ONH. In all cases, the schisis appeared as a localised, heterogeneous microcystic mass within the RNFL and sometimes also partially on top of it (Cases  4 and 5). In most cases, other layers under the RNFL were involved as well, such as the ganglion cell layer (Cases  1, 4, and 6), or even deeper retinal layers, like the inner and outer nuclear layer (Cases  3 and 5). None of the SD-OCT scans of these cases showed any signs of other ONH or retinal abnormalities, such as posterior vitreous detachment or a macular pucker. An overview with images of three cases is presented in Figure 1.

### 3.2. Outcome Other Imaging Devices

No other imaging device captured this localised retinoschisis as clearly as the SD-OCT did. In 6 cases, the visual field showed a glaucomatous defect in the sectors corresponding with the location of the schisis in the SD-OCT images. In the case without a glaucomatous visual field defect in the corresponding sector (case  7), the thickening was located in a very small wedge defect. In none of the cases was the RNFL schisis detectable by SLP (GDx); its image only showed a localised RNFL wedge at the same location. In three cases, the retinoschisis was displayed also by SLT (HRT-3D), presented as a slightly elevated surface in the three-dimensional image. In one case, we performed fluorescence angiography during the retinoschisis to seek for any signs of a pathophysiological mechanism. Besides some atherosclerotic arteries, the angiogram showed no pathological patterns, such as leakage or other signs of inflammation.

**Figure 2:**
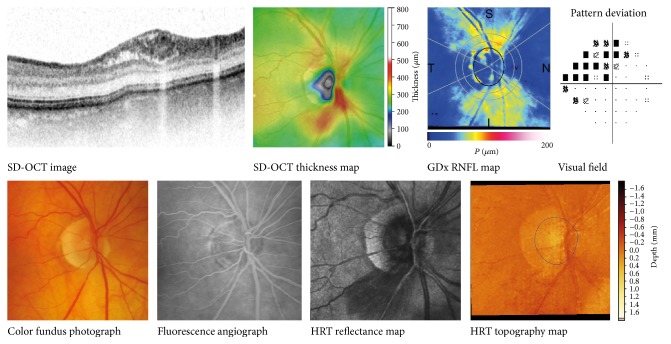
SD-OCT, scanning laser polarimetry (GDx), visual field, color fundus photograph, fluorescence angiograph, and scanning laser tomography (HRT) images during the presence of the focal retinoschisis in Case  6. The SD-OCT image shows the retinoschisis inferotemporally (see thickness map). The thickness map shows the total retinal thickness. The GDx shows no increased RNFL thickness. The color fundus photograph as well as the fluorescein angiography shows no clear signs of the retinoschisis, except for atherosclerotic arteries inferiorly to the ONH. The HRT shows a darker area at the thickened location in the reflectance map, as well as a slight increased height in the topography map. SD-OCT: spectral domain optical coherence tomography; RNFL: retinal nerve fiber layer.

The disagreements between the various imaging techniques in the ability to capture the transient, focal retinoschisis have been illustrated in Figure 2.

### 3.3. Outcome over Time

All 7 subjects were measured at least four times with SD-OCT at 6-monthly intervals. In Case  6 we saw that the retinoschisis was present at the fourth measurement and that it had disappeared after 13 months. In Cases  4 and 5, the retinoschisis disappeared, and in Cases  2 and 3, the schisis occurred within the time frame of these measurements. In Cases  1 and 7, we observed the retinoschisis in all measurements, suggesting it can last for at least 27 months. The images taken with and without the schisis, in the same location, have been presented in Figure 1.

Of the 5 eyes imaged both with and without the presence of retinoschisis, 4 eyes were imaged with other devices as well. As presented in Table 3, the defect in the visual field, expressed by the MRS, appeared to be worse during the time of retinoschisis. In 3 of these 4 eyes, the MRS decreased by at least 2.5 dB during the retinoschisis, statistically nonsignificantly however. In none of these 4 eyes did the GDx and HRT-TCA show any change in height. The HRT-3D image did reveal a focally elevated surface when the schisis was present.

## 4. Discussion

This study presents a focal schisis of the retinal nerve fiber layer (RNFL) and sometimes deeper retinal layers in glaucomatous eyes. None of the healthy eyes showed this phenomenon. In all cases, the retinoschisis was located in an area of a preexisting glaucomatous RNFL defect, which suggests that this phenomenon is associated with glaucomatous wedge defects of the RNFL.

Several recent studies presented cases with peripapillary retinoschisis, imaged with SD-OCT [8–13]. All studies showed a relation of the retinoschisis with the presence of glaucoma. We also observed the retinoschisis in glaucomatous eyes only, all verging on a glaucomatous (wedge) defect. Kahook et al. [11] presented two cases with retinal schisis cavities in the presence of narrow occludable angles and increased intraocular pressure, suggesting a tight relation, which has been confirmed by Lee et al. [12] Our data did not confirm this finding, but we found the retinoschisis in open angles and with low intraocular pressures as well, suggesting a different pathophysiology. Because they followed a large population for several years, Lee et al. were able to observe spontaneous resolution of the retinoschisis [12]. We also observed that the retinoschisis appeared and disappeared with various time spans between eyes, ranging from less than six months up to over 27 months.

None of the previously mentioned studies adopted other techniques commonly used for monitoring glaucoma to investigate their ability to detect this retinoschisis or to relate it to glaucomatous loss or potential progression. We followed all participants longitudinally with visual field testing, SLP, SLT, and SD-OCT. All these techniques measured a glaucomatous (wedge) defect at the location of the retinoschisis. Interestingly, the schisis itself of the RNFL and sometimes other deeper retinal layers could hardly be detected by any of the other imaging devices. SLP (GDx) did not detect this schisis at all. This is not very surprising because the GDx measures retardation. Without any change to the number of nerve fibers and without any substantial change in their course, we would not have expected any changes in retardation. The apparent accumulation of fluid within the schisis area would not have led to a change in retardation, similar to the lack of change in case of disc edema [17]. SLT (HRT) did capture the thickening in the three-dimensional surface display, but it failed to detect any schisis by the HRT parameters.

In the eyes in which the retinoschisis appeared between measurements or disappeared over time, a corresponding difference in RNFL thickness on SD-OCT was observed. The HRT and GDx did not appear to measure any differences in RNFL thickness between measurements prior to or after the RNFL schisis nor during its presence. The visual field, however, showed a, statistically not significant, decreased mean retinal sensitivity (MRS) of at least 2.5 dB during the retinoschisis at the exact location corresponding with the schisis [14], compared to the visual field before or after the retinoschisis. This suggests a temporary decreased function due to the retinoschisis, fortunately not leading to permanent progression of the glaucomatous defect.

The pathophysiological mechanism of this schisis is unclear. Most likely, the schisis contains fluid, since its reflectivity is lower than the surrounding tissues and it absorbs or scatters part of the incident light. The origin of the fluid remains unclear. Potentially, thickening and fluid accumulation might be characteristic of inflammation. The absence of other signs of inflammation, including the fully normal fluorescence angiography in our study, suggests another cause. As suggested by others [8, 11, 12], the fluid might be of vitreous origin. The fluid might enter the RNFL through microscopic interconnections, leading to the schisis. Kahook et al. hypothesize that pressure peaks lead to changes in axial length, which might lead to vitreous traction [11]. Whenever the vitreous detaches from the inner limiting membrane, this might lead to peripapillary vitreoretinal traction syndrome with retinal thickening next to the ONH [15, 16, 18]. Our SD-OCT scans, combined with retrospective medical chart search for any PVD observations, showed no signs of other ONH or retinal abnormalities potentially causing thickening and edema inside the retina. Since all cases differed somewhat in their treatment, we do not think that the retinoschisis was therapy related.

The focal retinoschisis always verged on the ONH, suggesting that it follows the pattern of loss of nerve fibers. In addition, the RNFL schisis seems to follow the nerve fibers into the lamina cribrosa, suggesting that the fluid might enter the RNFL from the subarachnoidal space through the lamina cribrosa along the tiny canals created by the pathological absence of previously present nerve fibers. Recently, Lee et al. strengthened this hypothesis by presenting the relationship between retinoschisis and damage to the lamina cribrosa [13]. They found that the location of the lamina cribrosa abnormalities, found on OCT with enhanced depth imaging, appeared to be associated with the involved retinal layers. Lee et al. related this hypothesis to the presence of an optic pit, which was found in 36% of their cases [12]. The eyes in our study did not show any optic pits. Interestingly, cerebrospinal fluid contains proteins, which in SD-OCT potentially lead to shadowing effects, causing relatively lower reflectivity of the retinal layers underneath. This matches our findings as illustrated in Figure 1, strengthening this hypothesis. Whether proteins would retain between or in the retinal layers after the resolution of the fluid remains unclear. Our data did not show any visible signs of differences in reflectivity of the retinal layer at the location of the former schisis and its surrounding area, suggesting that the proteins dissolve completely.

Our study was subject to some limitations. First, this study was based on a review of images obtained from a large prospective longitudinal study into glaucoma imaging, which was not specifically designed for investigating the clinical course of retinoschisis. Second, the sample size was small. We therefore think that the only remarkable finding in the patient characteristics, that is, that all subjects that showed the retinoschisis were women, is probably coincidental and due to this small sample size. On the other hand, a large group of subjects with glaucoma were included in our long-term follow-up study on glaucoma imaging, while only few showed the retinoschisis. In fact, only about 4% of the 189 eyes measured for many years showed the retinoschisis. Further longitudinal follow-up of a larger population is necessary to address the exact course of the retinoschisis and its potential relation to glaucomatous progression.

In conclusion, our data suggest that transient, localised, peripapillary schisis of the RNFL and sometimes other deeper retinal layers is associated with glaucomatous RNFL wedge defects. Of all techniques used to monitor glaucoma in daily practice, the retinoschisis was best observed with SD-OCT. The visual field showed a temporary focally decreased sensitivity during the retinoschisis. We found no evidence that the retinoschisis predicts glaucomatous progression.

## Figures and Tables

**Table 1:** Subject characteristics.

Case	Eye	Age (years)	IOP (mmHg)	Severity Glaucoma^*∗*^	Glaucoma treatment	Surgical interventions
#1	OD	61.5	14	Moderate	None	Trabeculectomy (2007)
#2	OD	63.2	15	Advanced	None	Trabeculectomy (1995)
#3	OD	76.2	12	Mild	Dorzolamide/Timolol; Latanoprost	None
#4	OD	72.8	14	Advanced	Dorzolamide/Timolol; Carteolol	None
#5	OD	61.8	12	Moderate	Timolol; Bimatoprost	Trabeculectomy (2000)
#6	OD	80.6	21	Mild	Timolol	YAG PI (1996), Trabeculoplasty (1999)
#7	OS	78.6	12	Mild	Timolol	YAG PI (1996)

^*∗*^Mild glaucoma: MD > −6 dB; moderate glaucoma −12 dB > MD > −6 dB; advanced glaucoma <−12 dB.

IOP: intraocular pressure; YAG PI: yttrium-aluminium-garnet (YAG) laser peripheral iridotomy; MD: Mean Deviation (of visual field).

**Table 2:** Characteristics of SD-OCT and visual field of each case.

Case	SD-OCT		VF
	Location RNFL schisis	Layers involved	Number of abnormal points
#1	Superotemporal (ST)	RNFL, GCL	10 (10) (ST)
#2	Superotemporal (ST)	RNFL, GCL	7 (10) (ST)
#3	Nasal (N)	RNFL, GCL, and ONL	1 (4) (N)
#4	Temporal (T + IT)	RNFL	17 (19) (T/IT)
#5	Inferotemporal (T + IT)	RNFL, GCL, and INL	7 (16) (T/IT)
#6	Inferotemporal (IT)	RNFL, GCL	8 (13) (IT)
#7	Superotemporal (ST)	RNFL	0 (10) (ST)

An abnormal point was an individual point below the 0.5% probability level in either the total or the pattern deviation plot. The number of abnormal points (+ total amount of points) was counted per sector corresponding with the location of the schisis of the RNFL in SD-OCT [14].

SD-OCT: spectral domain optical coherence tomography; RNFL: retinal nerve fiber layer; GCL: ganglion cell layer; ONL: outer nuclear layer; INL: inner nuclear layer; VF: visual field.

**Table 3:** Measurement characteristics of visual field of four cases of measurements with and without the focal retinoschisis.

Case	Visual field				
	MRS during retinoschisis	Moment without retinoschisis	MRS without retinoschisis	Difference	*p* value^*∗*^
#3	24.3 [SD 5.1]	<	21.3 [SD 3.3]	3.0	0.19
#4	5.5 [SD 9.1]	>	8.2 [SD 10.9]	−2.7	0.04
#5	17.6 [SD 9.4]	>	20.1 [SD 9.5]	−2.5	0.14
#6	17.3 [SD 7.0]	< & >	20.9 [SD 6.6]	−3.5	0.04

The MRS was calculated in the sector corresponding with the location of the RNFL schisis on spectral domain optical coherence tomography [14]. The moment without retinoschisis is presented as before (<) or after (>) the retinoschisis.

^*∗*^Paired Student's *t*-test.

MRS: mean retinal sensitivity; RNFL: retinal nerve fiber layer.

## References

[1] Quigley H. A., Nickells R. W., Kerrigan L. A., Pease M. E., Thibault D. J., Zack D. J. (1995). Retinal ganglion cell death in experimental glaucoma and after axotomy occurs by apoptosis. *Investigative Ophthalmology and Visual Science*.

[2] Ritch R., Shields M. B., Krupin Th. (1996). Anatomy and pathophysiology of the retina and optic nerve. *The Glaucomas; Basic Sciences*.

[3] Lin S. C., Singh K., Jampel H. D. (2007). Optic nerve head and retinal nerve fiber layer analysis. a report by the American Academy of Ophthalmology. *Ophthalmology*.

[4] Cense B., Nassif N. A., Chen T. C. (2004). Ultrahigh-resolution high-speed retinal imaging using spectral-domain optical coherence tomography. *Optics Express*.

[5] Nassif N. A., Cense B., Park B. H. (2004). In vivo high-resolution video-rate spectral-domain optical coherence tomography of the human retina and optic nerve. *Optics Express*.

[6] Nassif N., Cense B., Park B. H. (2004). *In vivo* human retinal imaging by ultrahigh-speed spectral domain optical coherence tomography. *Optics Letters*.

[7] Wojtkowski M., Leitgeb R., Kowalczyk A., Bajraszewski T., Fercher A. F. (2002). In vivo human retinal imaging by Fourier domain optical coherence tomography. *Journal of Biomedical Optics*.

[8] Bayraktar S., Cebeci Z., Kabaalioglu M., Ciloglu S., Kir N., Izgi B. (2016). Peripapillary retinoschisis in glaucoma patients. *Journal of Ophthalmology*.

[9] Farjad H., Besada E., Frauens B. J. (2010). Peripapillary schisis with serous detachment in advanced glaucoma. *Optometry and Vision Science*.

[10] Hwang Y. H., Kim Y. Y., Kim H. K., Sohn Y. H. (2014). Effect of peripapillary retinoschisis on retinal nerve fibre layer thickness measurement in glaucomatous eyes. *British Journal of Ophthalmology*.

[11] Kahook M. Y., Noecker R. J., Ishikawa H. (2007). Peripapillary schisis in glaucoma patients with narrow angles and increased intraocular pressure. *American Journal of Ophthalmology*.

[12] Lee E. J., Kim T.-W., Kim M., Choi Y. J. (2014). Peripapillary retinoschisis in glaucomatous eyes. *PLOS ONE*.

[13] Lee J. H., Park H. L., Baek J., Lee W. K. (2016). Alterations of the lamina cribrosa are associated with peripapillary retinoschisis in glaucoma and pachychoroid spectrum disease. *Ophthalmology*.

[14] Garway-Heath D. F., Poinoosawmy D., Fitzke F. W., Hitchings R. A. (2000). Mapping the visual field to the optic disc in normal tension glaucoma eyes. *Ophthalmology*.

[15] Hedges T. R., Flattem N. L., Bagga A. (2006). Vitreopapillary traction confirmed by optical coherence tomography. *Archives of Ophthalmology*.

[16] Hixson A., Reynolds S. (2011). Peripapillary vitreoretinal traction. *Optometry*.

[17] Banks M. C., Robe-Collignon N. J., Rizzo J. F., Pasquale L. R. (2003). Scanning laser polarimetry of edematous and atrophic optic nerve heads. *Archives of Ophthalmology*.

[18] Kroll P., Wiegand W., Schmidt J. (1999). Vitreopapillary traction in proliferative diabetic vitreoretinopathy. *British Journal of Ophthalmology*.

